# Feasibility and effectiveness of multi-injection thoracic paravertebral block via the intrathoracic approach for analgesia after thoracoscopic-laparoscopic esophagectomy

**DOI:** 10.1007/s10388-020-00807-9

**Published:** 2021-01-06

**Authors:** Lihong Hu, Xia Xu, Weiyu Shen, Jinxian He

**Affiliations:** 1 Department of Anesthesiology, Ningbo Medical Center Lihuili Hospital, Ningbo, 315040 China; 2 Department of Thoracic Surgery, Ningbo Medical Center Lihuili Hospital, Ningbo, 315040 China

**Keywords:** Thoracic paravertebral block, Thoracoscopic-laparoscopic esophagectomy, Postoperative analgesia, Multi-injection

## Abstract

**Background:**

We observed the feasibility and effectiveness of multi-injection thoracic paravertebral block (TPB) via the intrathoracic approach under thoracoscopic direct vision for analgesia after thoracoscopic-laparoscopic esophagectomy (TLE).

**Methods:**

Sixty patients undergoing TLE were randomly divided into a control group and an observation group. All patients underwent TPB via the intrathoracic approach at the three levels of T2, 5, and 8 with a scalp needle before closing the chest. The patients in the observation group received 10 ml 0.375% ropivacaine at each level, and the patients in the control group received 10 ml of 0.9% saline at each level. A patient-controlled intravenous analgesic (PCIA) pump with sufentanil was attached to all patients after surgery. The sufentanil consumption, number of PCIA presses and use of rescue analgesia in the first 24 h after surgery were recorded. The visual analogue scale (VAS) scores (rest and coughing) were recorded at 2 h, 6 h, 12 h, 24 h, and 48 h after surgery. The duration of postoperative hospital stay, active cough rate, first ambulation, and the incidence of adverse reactions after surgery was recorded.

**Results:**

The sufentanil consumption in the observation group was significantly lower than that in the control group (34.7 ± 1.9 µg vs. 52.1 ± 2.1 µg; *P* < 0.001). The VAS score at each postoperative time point, number of PCIA presses, use of rescue analgesia, and the incidence of adverse reactions in the observation group were significantly lower than those in the control group. The postoperative active cough rate of patients in the observation group was significantly higher than those in the control group, and the times of the first ambulation after surgery and postoperative hospital stay in the observation group were significantly shorter than those in the control group (all *P* < 0.05).

**Conclusions:**

Multi-injection TPB via the intrathoracic approach under thoracoscopic direct vision is easy to perform and can effectively alleviate postoperative pain after TLE with fewer adverse reactions and contributing to improved postoperative recovery.

**Supplementary Information:**

The online version contains supplementary material available at 10.1007/s10388-020-00807-9.

## Introduction

Esophageal cancer is one of the most common malignant tumors both in China and the world [1], and surgical treatment is the first choice [2]. Radical resection is the main clinical treatment for esophageal cancer. Traditional open esophagectomy results in severe trauma and pain, as well as a high incidence of complications and a long hospital stay [3]. The minimally invasive thoracoscopic radical esophagectomy has the advantages of less trauma, faster recovery, and fewer complications, and has been used increasingly in recent years [4]. Thoracoscopic-laparoscopic esophagectomy (TLE) is completely minimally invasive surgery [5]. The TLE surgery is extensive, involving three areas, the neck, chest, and abdomen. The postoperative pain is, therefore, still severe, which seriously affects the postoperative recovery. The effective reduction of postoperative pain after TLE is an important focus of the surgical team.

In recent years, the thoracic paravertebral nerve block (TPB) technique has been applied to postoperative analgesia for esophageal cancer. At present, the commonly-used TPB technique places the catheter in the extra-pleural paravertebral space via percutaneous puncture guided by ultrasound or direct vision using thoracoscopy. Local anesthetic (LA) is then diffused in the paravertebral space 2–4 thoracic vertebral levels above and below the injection site [6, 7]. TPB can effectively block the sensory, motor, and sympathetic nerve fibers of the thoracic segment, producing a unilateral epidural block. Ultrasound-guided TPB, however, has the disadvantages of technical difficulty, necessity for working in a limited operating space, and a low success rate. Catheter implantation through a percutaneous puncture is complex and difficult and carries the risk of puncturing the pleura and damaging the intercostal nerves or sympathetic chain. These methods use a single site block which limits the range of the block and does not cover the thoraco-abdominal surgical field of TLE. Our previous study found that before the end of a single-port thoracoscopic lobectomy, a single injection of 0.375% ropivacaine in the thoracic 4 paravertebral space under thoracoscopic direct vision could achieve the necessary paravertebral block, effectively reducing postoperative pain and adverse reactions, and thus, being conducive to postoperative recovery [8]. This raised the question of the possibility of direct injection of LA into multiple paravertebral spaces using the intrathoracic approach under thoracoscopic direct vision. The aim was to implement an extensive paravertebral block to achieve a high degree of paravertebral nerve block covering the surgical fields of TLE, and ultimately to reduce postoperative pain. There are no previous reports on the application of multiple-injection TPB via the intrathoracic approach in thoracic surgery. Therefore, this study aimed to investigate the feasibility and effectiveness of multiple injections of TPB via the intrathoracic approach under thoracoscopic direct vision to improve the postoperative analgesia after TLE.

## Patients and methods

### Patients

This study is a randomized double-blind controlled study. The protocol was approved by the Ethics Committee of Ningbo Medical Center, Lihuili Hospital, China (approval reference KY2020PJ015). The study was registered with the Chinese Clinical Trial Registry (number ChiCTR2000034726). Sixty patients undergoing TLE under general anesthesia were enrolled between March 2020 and September 2020 in the Ningbo Medical Center Lihuili Hospital. The inclusion criteria for patients were as follows: age between 50 and 79 years, the preoperative staging of I–II for esophageal cancer, an American Society of Anesthesiologists (ASA) physical status of I–III, and a requirement for minimally invasive surgery. The exclusion criteria were patients with a spinal deformity or spinal surgical history, allergies to LAs, and patients who did not consent to the procedure. All patients signed informed consent.

The patients were randomly and equally divided into a control group and an observation group using a computer-generated random table.

### Anesthesia

Standard monitoring was performed when the patients entered the operating theater. Rapid intravenous induction with 0.05 mg kg^−1^ midazolam, 2 mg kg^−1^ propofol, 0.2 μg kg^−1^ sufentanil, and 0.6 mg kg^−1^ rocuronium was performed. After tracheal intubation, a bronchial occluder was optimally positioned using fiberoptic bronchoscope guidance. Intermittent positive pressure ventilation was given to ensure a tidal volume of 6 ml kg^−1^, and a respiratory rate of 10–12 times min^−1^. The end-tidal carbon dioxide (P_ET_CO_2_) was maintained between 35 and 40 cmH_2_O during the surgery. After anesthesia induction, the left radial artery and the right internal jugular vein puncture were placed. The intraoperative anesthesia maintenance was 8 mg·kg^−1^·h^−1^ propofol, 0.1 μg·kg^−1^·min^−1^ remifentanil, and 0.2 mg (kg·0.5 h) rocuronium with a single intravenous injection.

### Surgery

**Fig. 1:**
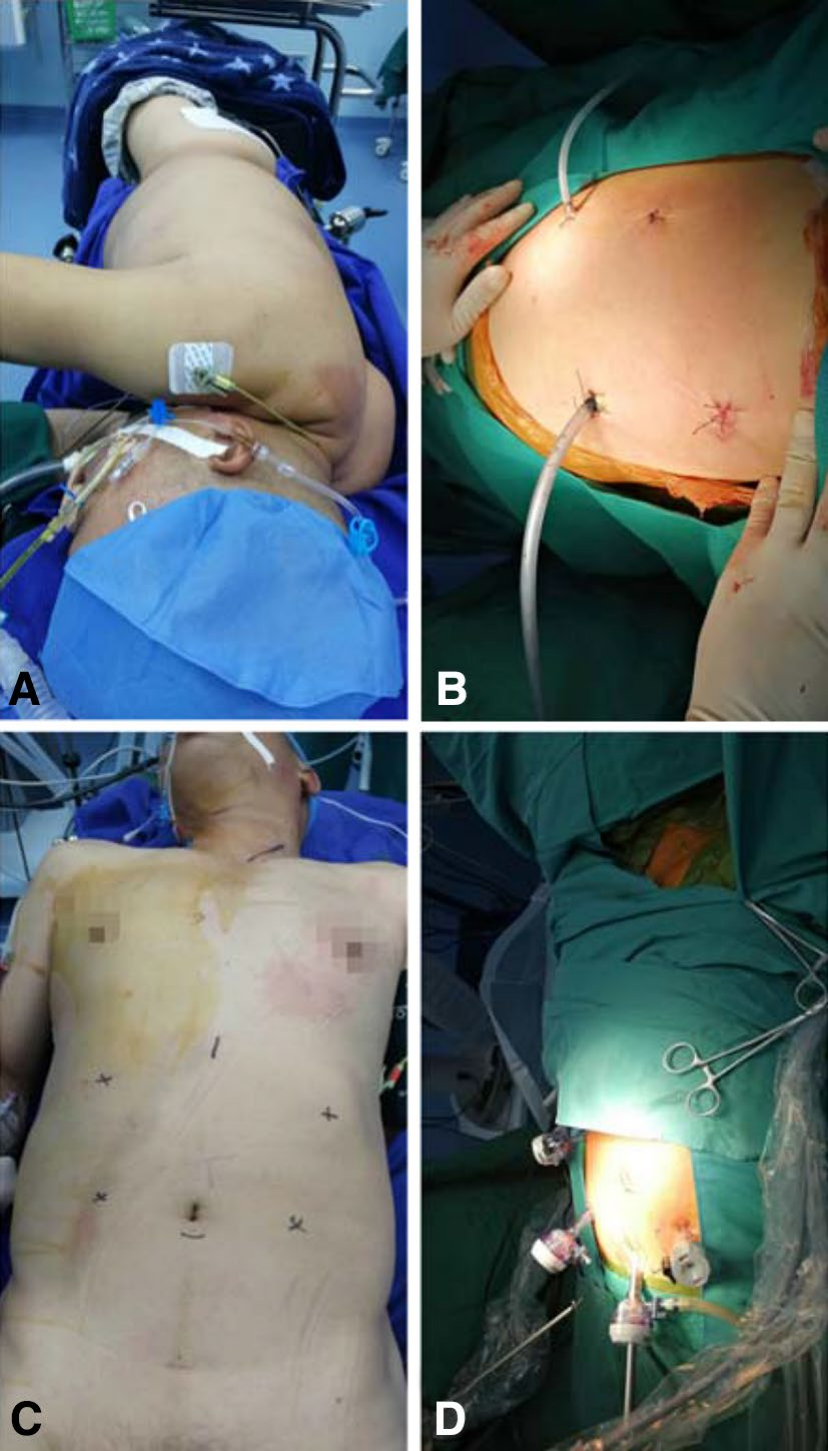
Intraoperative position and incision of the surgery. **a** Left-side decubitus position. **b** The incision and port location of thoracoscopic surgery. **c** Patient was placed in a supine position. The abdominal and neck incision marking. **d** The incision and port location of laparoscopic surgery

The two groups of patients underwent TLE through three areas: the right chest, upper abdomen, and the left neck. The patient was laid on the operating table in the left-side decubitus position. During the surgery, the operating table was tilted to the left according to requirements, so that the patient was in a left-inclined position about 135 degrees from the horizontal plane (Fig. 1a). The first was the four-ports thoracoscopic surgery (Fig. 1b). After the thoracic surgery, the patient was placed in a supine position, with the head tilted to the right (Fig. 1c). The abdomen and neck surgeries were started after re-disinfecting. Four-ports laparoscopic surgery was used on the abdomen and a small incision on the left neck (Fig. 1d).

### Analgesia methods

**Fig. 2:**
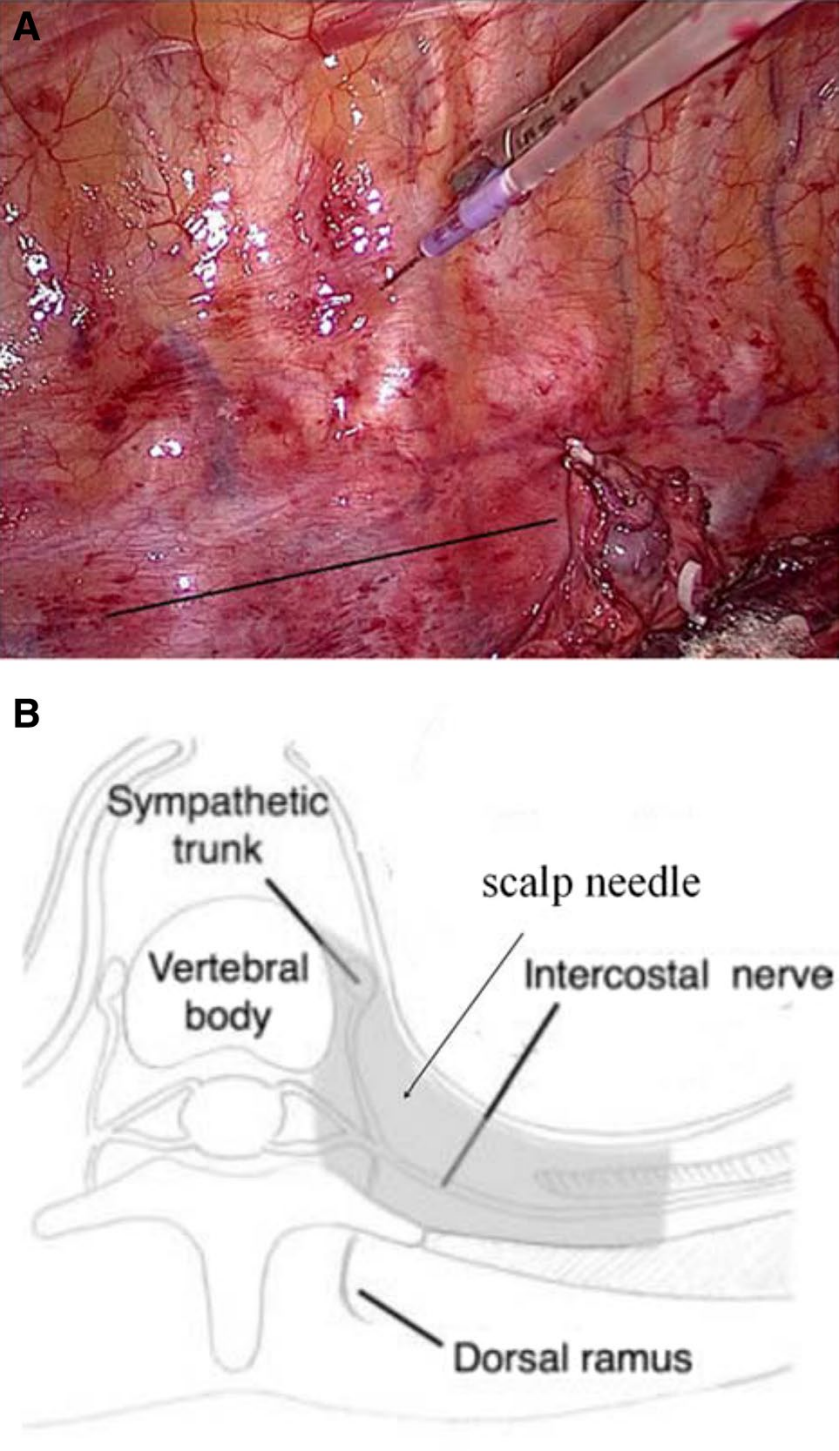
Thoracic paravertebral block (TPB) during the surgery. **a** TPB at T5 level via the intrathoracic approach under thoracoscopic direct vision. The black line shows the thoracic vertebrae. **b** Horizontal plane of paravertebral space: the gray area represents paravertebral space [9] and site of scalp needle puncture

In the observation group, a scalp needle with an extended tube was inserted into the paravertebral space at the T2, 5, and 8 levels under thoracoscopic direct vision (Fig. 2a). A total of three sites each 1 cm adjacent to the vertebrae were inserted vertically 0.5 cm under the parietal pleura with the needle, and 10 ml 0.375% ropivacaine was injected at each site (Fig. 2b). No hemorrhage or hematoma was observed after five minutes. The control group was injected with the same volume of normal saline at each site. The two groups were given 0.2 µg kg^−1^ sufentanil and 2 mg tropisetron half an hour before the end of surgery. At the end of the surgery, the PCIA pump was connected to patients to administer 1.5 µg·kg^−1^ sufentanil and 5 mg tropisetron diluted to 100 ml with normal saline. The parameters were set as a continuous dose of 2 ml h^−1^, a bolus dose of 1 ml, and a locking time of 15 min. When the VAS score was greater than five or the pain was unbearable, 40 mg parecoxib was injected intramuscularly.

Our study used pilot testing to compare the sufentanil consumption within the first 24 h after surgery between the control and observation groups. Pilot testing using ten patients showed a mean [standard deviation] sufentanil consumption of 38.4 [4.75] μg 24 h after surgery in the observation group and 44.5 [5.75] μg in the control group. The requirement was 26 patients (13 in each group), which was calculated by the MedSci Sample Size Tools at a power of 0.8 with 0.05 alpha. Thus, to compensate for the possibility of missing data or dropouts, we recruited 30 patients for each group.

### Data collection

The primary outcome was the sufentanil consumption infused by the PCIA in the first 24 h after surgery. The secondary outcome was the VAS during rest and while coughing at the time points of 2 h, 6 h, 12 h, 24 h, and 48 h after surgery. The number of patients that required intravenously injected parecoxib for rescue analgesia and number of PCIA presses in the first 24 h after surgery were recorded. The duration of postoperative hospital stay, active cough rate, first ambulation and the incidence of adverse reactions after surgery were recorded.

### Statistical analysis

Data were analyzed using SPSS version 23.0 (IBM Corp. Armonk, NY, USA). Continuous variables conforming to the features of normal distribution were represented as mean ± standard deviation and analyzed using the *t *test. Repeated measurement data were analyzed using repeated ANOVA. Variables with skewed distribution were presented as medians (quartiles) and compared using the Kruskal–Wallis *H* test. Qualitative variables were presented as numbers (percentage) and analyzed using the chi-square (*χ*^2^) test. The difference was statistically significant with *P* < 0.05.

## Results

**Fig. 3:**
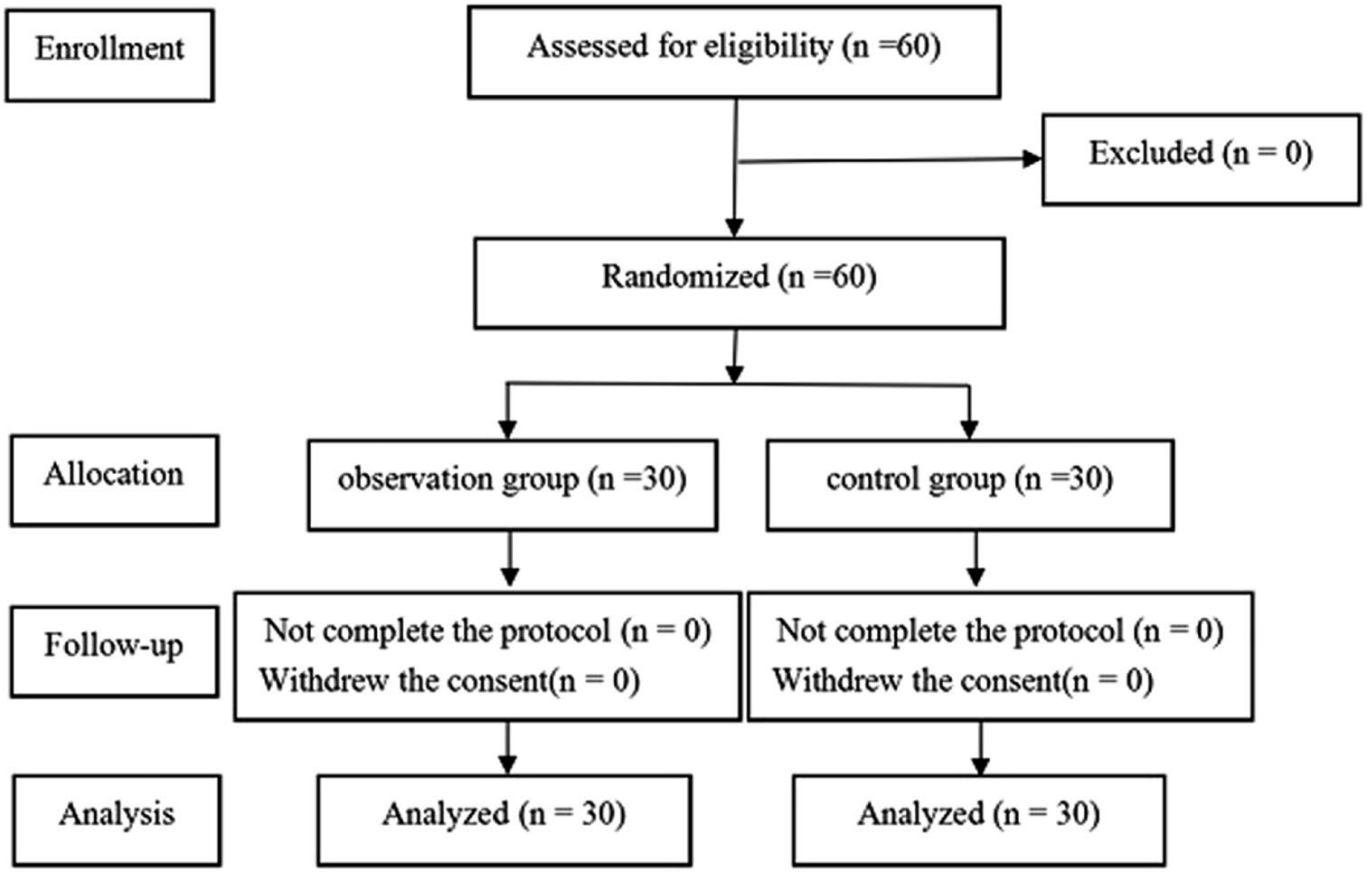
Patient flow diagram

A total of 60 patients were included and completed the study. All patients had successfully performed TLE. The Consolidated Standards of Reporting Trials (CONSORT) diagram is shown in Fig. 3. The patients’ characteristics and surgical parameters are shown in Table 1. There were no significant differences between the two groups.

**Table 1:** Characteristics and surgical parameters

	Control group	Observation group	*P* value
Number of patients	30	30	
Gender			1
Male	26 (86.7%)	27 (90.0%)	
Female	4 (13.3%)	3 (10.0%)	
Age (years)	65.0 ± 6.2	65.2 ± 6.2	0.900
ASA status			0.962
I	20 (66.7%)	19 (63.4%)	
II	9 (30.0%)	10 (33.3%)	
III	1 (3.3%)	1 (3.3%)	
Staging of cancer			0.795
I	17 (56.7%)	16 (53.3%)	
II	13 (43.3%)	14 (46.7%)	
Smoking history	20 (66.7%)	21 (70.0%)	0.781
Weight (kg)	66.4 ± 7.3	67.1 ± 6.1	0.688
Surgery time (min)	311.7 ± 22.7	313.1 ± 20.4	0.803
Amount of bleeding (ml)	218.4 ± 19.0	220.6 ± 20.6	0.669

Data are presented as mean ± standard deviation or number (%)

*ASA* American Society of Anesthesiologists

### Primary outcome

**Fig. 4:**
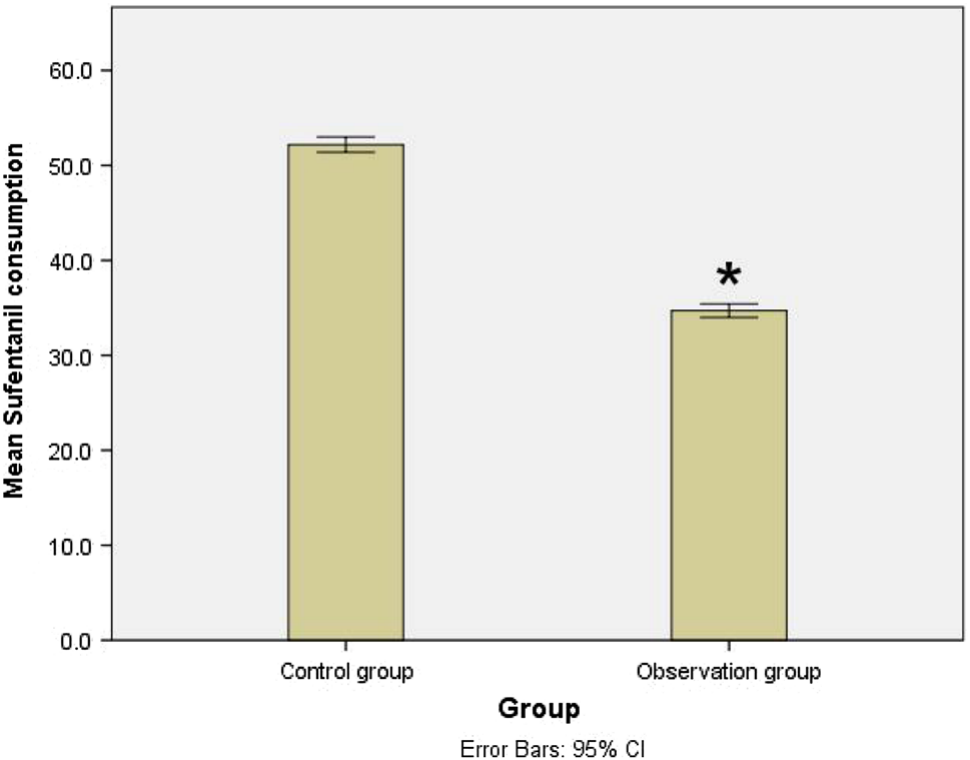
Sufentanil consumptions within the first 24 h after surgery of the observation group and control group. *P < 0.001

The sufentanil consumption within the first 24 h after surgery in the observation group was significantly lower than that in the control group (34.7 ± 1.9 µg vs. 52.1 ± 2.1 µg; *P* < 0.001) (Fig. 4).

### The second outcome

The VAS scores during rest and coughing in the observation group were significantly lower than those in the control group at 2 h, 6 h, 12 h, 24 h, and 48 h after surgery (Table 2). The VAS scores during rest and coughing at 48 h after surgery were significantly higher than at the time points of 2 h, 6 h, 12 h, and 24 h after surgery in the observation group, while the VAS scores 48 h after surgery were also significantly higher than the time points of 2 h, 6 h, 12 h, and 24 h after surgery in the control group.

**Table 2:** The VAS score after surgery

		Control group (*n* = 30)	Observation group (*n* = 30)	*P* value
During rest	2 h after surgery	4.5 ± 0.4	2.6 ± 0.3	< 0.001*
	6 h after surgery	4.8 ± 0.3	2.5 ± 0.2	< 0.001*
	12 h after surgery	4.6 ± 0.3	2.4 ± 0.2	< 0.001*
	24 h after surgery	4.4 ± 0.4	2.3 ± 0.2	< 0.001*
	48 h after surgery	5.2 ± 0.3	3.1 ± 0.3	< 0.001*
	*F* value	27.4	203.3	
	*P* value	< 0.001^#^	< 0.001^#^	
While coughing	2 h after surgery	4.9 ± 0.4	3.1 ± 0.3	< 0.001*
	6 h after surgery	5.3 ± 0.4	3.0 ± 0.3	< 0.001*
	12 h after surgery	5.1 ± 0.4	3.0 ± 0.3	< 0.001*
	24 h after surgery	4.8 ± 0.4	3.0 ± 0.3	< 0.001*
	48 h after surgery	5.6 ± 0.3	4.0 ± 0.3	< 0.001*
	*F* value	145.9	1300.8	
	*P* value	< 0.001^#^	< 0.001^#^	

Data are presented as mean ± standard deviation

*P < 0.001 Observation group vs Control group; ^#^*P* < 0.001 48 h after surgery vs 2 h, 6 h, 12 h, and 24 h after surgery

The observation group pressed the PCIA pump (0 [0–0] times) less than the control group (3.5 [2–4.25] times) (*P* < 0.001). The rates of rescue analgesia within the first 24 h after surgery were significantly lower than those in the control group (Table 3).

**Table 3:** The number of PCIA presses and use of rescue analgesia within the first 24 h after surgery

	Control group (*n* = 30)	Observation group (*n* = 30)	*P* value
Number of PCIA presses (times)	3.5 (2–4.25)	0 (0–0)	< 0.001*
Rescue analgesia (%)	7 (23.3%)	1 (3.3%)	0.016*

Data are presented as median (quartiles) or number (%)

*P < 0.05

The incidence of postoperative adverse reactions such as nausea, vomiting, pneumonia, hypoxemia, and atelectasis in the observation group were significantly lower than those in the control group. No significant differences in the incidences of pruritus, somnolence, and anastomotic leakage were observed between the two groups (Table 4).

**Table 4:** The incidence of adverse reactions after surgery

	Control group (*n* = 30) (%)	Observation group (*n* = 30) (%)	*P* value
Nausea	10 (33.3.%)	2 (6.7%)	0.010*
Vomit	8 (26.7%)	1 (3.3%)	0.030*
Pruritus	4 (13.3%)	0 (0.0%)	0.121
Somnolence	3 (10.0%)	0 (0.0%)	0.236
Pneumonia	10 (33.3%)	3 (10.0%)	0.028*
Hypoxemia	7 (23.3%)	0(0.0%)	0.016*
Atelectasis	8 (26.7%)	1 (3.3%)	0.030*
Anastomotic leakage	0 (0.0%)	0 (0.0%)	1

Data are presented as number (%)

*P* < 0.05

The active cough rates of the observation group were significantly higher than those of the control group. Both the first ambulation after surgery and the postoperative hospital stay were significantly shorter in the observation group than those in the control group (Table 5).

**Table 5:** The postoperative recovery

	Control group (*n* = 30)	Observation group (*n* = 30)	*P* value
Active cough rate (%)	3 (10.0%)	10 (33.3%)	< 0.001*
First ambulation (h)	39.9 ± 2.6	30.0 ± 1.5	< 0.001*
Postoperative hospital stay (days)	11.1 ± 1.6	9.9 ± 1.3	0.002*

Data are presented as mean ± standard deviation or number (%)

*P < 0.05

## Discussion

China is one of the countries with a high incidence of esophageal cancer. By 2015, the incidence of esophageal cancer in China had risen to third place among all malignant tumors, with the mortality rate ranking fourth, and is, therefore, a major health issue [10]. Surgery is the first choice for treating early- and middle-stage esophageal cancer [1]. Traditional open surgery causes severe damage to the physical structure of the chest and abdomen walls, resulting in significant trauma, complications, and long periods of hospitalization [2]. The complications are mainly related to postoperative pain. Patients undergoing thoracic surgery have decreased postoperative lung function reserves. The severe and long-lasting pain after surgery prevents patients from breathing deeply with active coughing and sputum production, resulting in the retention of respiratory secretions, causing hypoxemia, atelectasis, lung infections, and even respiratory failure [11]. Moreover, pain causes severe stress responses and suppresses the immune function [12], while also restricting early ambulation and resumption of eating, both of which affect postoperative recovery [13]. Acute postoperative pain that is not well-managed can easily turn into postoperative chronic pain, seriously affecting the patients’ quality of life [14].

Recently, with the popularization of the concept of enhanced recovery after surgery and the development of minimally invasive techniques, endoscopic minimally invasive esophagectomy has been increasingly adopted [15]. TLE is completely minimally invasive surgery [5], with the advantages of less trauma, less bleeding, fewer complications, and rapid recovery, and is gradually replacing the traditional open surgery [16]. The surgery is performed through three incisions on the right chest, abdomen, and left neck, respectively [7]. The scope of the surgery is relatively large. Although the surgical incision is small and the trauma is reduced, the postoperative pain is still severe and there is also a high risk of complications, which seriously affects surgical effectiveness and postoperative recovery.

Adequate postoperative analgesia is conducive to early active coughing and sputum discharge, which improve lung function, reduce postoperative pulmonary complications, and promote postoperative recovery. Therefore, good postoperative analgesia has a positive significance for patients' rapid recovery and perioperative safety [17]. Currently, postoperative analgesia after esophagectomy mainly includes thoracic epidural analgesia, PCIA, and TPB [15]. Thoracic epidural analgesia is still the gold standard for postoperative analgesia after thoracic surgery. It is superior to PCIA with systemic opioids in both postoperative pain control and the reduction of pulmonary complications [18]. Nevertheless, it has disadvantages and complications, including (1) failure of the puncture or catheter placement, epidural perforation, catheter displacement, or obstruction; (2) epidural hematoma or abscess and nerve damage; (3) postoperative hypotension which can reduce the blood flow of the gastric duct, potentially leading to anastomotic leakage or gastric duct necrosis; (4) epidural operations cannot be performed in patients with abnormal blood coagulation, spinal deformities, or a history of spinal surgery, all of which limit its clinical application [19–21]. The block plane of the conventional single-point puncture has difficulty in covering the scope of TLE, and the analgesia is not perfect. While PCIA is simple and convenient for postoperative care [22], it requires large amounts of systemic opioids, with the risk of multiple adverse reactions such as nausea, vomiting, and even respiratory depression, and the early postoperative analgesic effect is not ideal [23].

Thoracic paravertebral nerve block is a technique of injecting LAs into the paravertebral space. It had been widely used in postoperative analgesia after thoracic surgery in recent years [24]. The thoracic paravertebral space is triangular in all three dimensions, containing spinal nerves, intercostal nerves, and sympathetic nerve chains from the intervertebral foramen [9]. The LAs spread in the paravertebral space of the two to four upper and lower segments of the injection site, so can block both the intercostal nerve and the sympathetic nerve at the same time [24]. This produces a similar effect to unilateral epidural block and can effectively reduce postoperative incision pain, catheter irritation pain, and visceral pain. At the same time, it can also avoid the hypotension caused by thoracic epidural analgesia which is widely used in clinical practice [25].

At present, there are two principal TPB methods. The first consists of a single injection block of LAs into the paravertebral space while, for continuous block, the catheter is placed in the paravertebral space percutaneously under ultrasound guidance [6]. However, due to the narrowness of the paravertebral space, limited operating space, and higher requirements for ultrasound block technology, there is a high failure rate. The second method is to place the catheter in the paravertebral space through percutaneous puncture outside the pleura under thoracoscopic direct vision, this can achieve a similar continuous block as the catheter placed under ultrasound guidance. TPB can effectively reduce both the postoperative pain and consumption of opioids after thoracic surgery [7, 24, 26, 27]. Both TPBs require at least three to five minutes during the procedure, possibly longer if the action is not smooth. The puncture outside the pleurapleural carries a risk of injury to the intercostal nerve and artery during the catheter placement. However, the LA spreads between two to four vertebral segments above and below the injection site in the paravertebral space. The degree of spread is positively correlated with the capacity of the LA. The block range cannot completely cover the TLE and the analgesic effect is not perfect.

The question of whether there is a simpler, faster, and less traumatic block method that can meet the needs of postoperative analgesia after TLE may be asked. The thoracic vertebrae and paravertebral structures are fully exposed under thoracoscopy. Considering the characteristics of thoracic paravertebral anatomy, the location of the nerve block, drug diffusion, and block plane, together with the method of the percutaneous puncture catheter implantation of the paravertebral block under thoracoscopy, it is important to determine whether LAs be directly injected into the paravertebral space through thoracoscopic direct vision to achieve the purpose of paravertebral block. Our previous study utilized injection of 20 ml 0.375% ropivacaine into the T4 level and 1 cm adjacent to the vertebrae under the thoracoscopic direct vision before the end of the single-port thoracoscopic lobectomy. Diffusion of the local anesthetic was found to induce paravertebral block, suggesting that this procedure could effectively reduce both postoperative pain and the risk of adverse reactions, contributing to successful postoperative recovery [8].

In this study, we used multi-injection TPB at the T2, 5, and 8 levels. After the separation of the esophagus had been completed and the chest tube had been placed in the chest, the scalp needle with the extended tube was inserted into the paravertebral space at the three levels (T2, 5, and 8) under thoracoscopic direct vision in the proper sequence. A total of three sites each 1 cm adjacent to the thoracic vertebrae were inserted vertically 0.5 cm under the parietal pleura and 10 ml 0.375% ropivacaine was injected into each. The sufentanil consumption, VAS score, number of PCIA presses, and the need for rescue analgesia in the observation group were significantly lower than in the control group. This demonstrates conclusively that the postoperative pain of patients in the observation group was significantly reduced, indicating that multi-injection TPB via the intrathoracic approach under thoracoscopic direct vision is feasible and effective. The block range of multi-injection TPB at the T2, 5 and 8 levels covered the right T1–T11 spinal nerve distribution area according to the diffusion characteristics of the LA in paravertebral block. These are the thoracic and abdominal surgical fields of TLE. TPB effectively alleviated postoperative pain and reduced the consumption of intravenous opioids. This was consistent with the findings of Zhang et al. [28]. The duration of the single-injection TPB was related to the concentration of the LA, generally within 12–24 h. The VAS score of the observation group at 48 h after surgery was significantly higher than that at 2 h, 6 h, 12 h, and 24 h after surgery. This also showed that the effect of single-injection TPB occurred within 24 h. The VAS score of the observation group at 48 h after surgery was significantly lower than that of the control group. This might be due to the following reasons: (1) The infusion time of the PCIA was set at 48–50 h, and the PCIA was still effective over this period in the observation group, while the analgesic drugs were used up in advance by repeated pressing in the control group. (2) TPB blocked the transmission of injurious stimuli to the central nervous system at the spinal level and activated the endogenous analgesia system. (3) The paravertebral block gradually subsided, increasing the patients’ pain tolerance threshold, to avoid pain hypersensitivity. This also suggested that even though the effect of the single TPB had diminished, it may still promote the alleviation of postoperative pain through other means.

Thoracoscopy has a magnifying function and the thoracic vertebrae and paravertebral structures were fully exposed after lung atrophy. The vertical distance between the parietal pleura and the intervertebral foramen is about 1.0 cm in adults. We chose the transmural pleura to insert the needle vertically at a depth of 0.5 cm to avoid the risk of damaging the nerve root, entering the spinal canal, and inducing general spinal anesthesia. Multi-injection TPB via the intrathoracic approach under thoracoscopic direct vision is easy to operate. It only requires checking the CT image structure of the block site before the operation and it can be completed quickly by the surgeon before closing the chest. The left decubitus position is used in thoracoscopic surgery, which is conducive to the manipulation of multi-injection TPB via intrathoracic approach under thoracoscopic direct vision. Other surgeons or institutions performed surgery in the prone position. We had also successfully implemented multi-injection TPB via intrathoracic approach under thoracoscopic direct vision in the prone position, and the procedure was the same as the left decubitus position.

The incidence of nausea and vomiting in the observation group was significantly lower than that in the control group, which may be due to less sufentanil consumption. The incidence of postoperative active cough in the observation group was significantly higher than that in the control group, and the incidences of pneumonia, hypoxemia, and atelectasis were significantly lower than those of the control group. Because the analgesic effect was good, chest, abdominal wall, and visceral pain, together with chest tube irritation caused by deep breathing were suppressed. The patients were confident about breathing, coughing, and sputum expectoration, all of which were beneficial to the recovery of postoperative respiratory function and avoided the corresponding respiratory system complications.

The time of the first ambulation after surgery and the duration of the postoperative hospital stay in the observation group were lower than those of the control group. This suggests that the postoperative recovery of the control group was better than that of the control group. This might be due to the following reasons: (1) The observation group used TPB, which had better postoperative analgesia and reduced opioid-related complications. (2) Patients were able to cough, expectorate, and breathe deeply, which helped to reduce postoperative pneumonia and other respiratory complications [10]. (3) Patients were able to exercise and get out of bed to further promote the recovery of respiratory functions, thus avoiding the possibility of deep vein thrombosis formation. (4) Good postoperative analgesia might inhibit the severe stress response caused by pain and thus may improve immune function [4]. (5) It benefits the early resumption of eating and gastrointestinal function recovery.

This study could only cover the area innervated by the right thoracic nerve. The pain of the left neck incision and the left abdomen could not be suppressed, indicating that additional analgesia methods are still needed. As this study did not investigate continuous block, the block effect might be shorter than a continuous block with catheter placement, either ultrasound-guided or thoracoscopic-guided.

In conclusion, multi-injection thoracic paravertebral block via the intrathoracic approach under thoracoscopic direct vision is simple and easy to perform and has been shown to effectively reduce pain after thoracoscopic-laparoscopic esophagectomy, with fewer adverse reactions and improved postoperative recovery.

## Supplementary Information

Below is the link to the electronic supplementary material.

Supplementary file1 (MP4 30763 KB)
